# A case of autosomal dominant polycystic kidney disease with systemic lupus erythematosus developing after SARS-CoV-2 vaccination

**DOI:** 10.1007/s13730-025-00999-y

**Published:** 2025-05-20

**Authors:** Kensuke Miyauchi, Joichi Usui, Tatsuya Shimizu, Akihisa Hattori, Soichiro Nomura, Takanobu Higashi, Toshiaki Usui, Chie Saito, Hirayasu Kai, Kunihiro Yamagata

**Affiliations:** 1 Department of Nephrology, University of Tsukuba Hospital, 2-1-1 Amakubo, Tsukuba, Ibaraki 305-8576 Japan; 2 Department of Nephrology, Institute of Medicine, University of Tsukuba, 1-1-1 Tennodai, Tsukuba, Ibaraki 305-8575 Japan; 3 Department of Nephrology, Ibaraki Prefectural Central Hospital, Ibaraki, 6528 Koibuchi, Kasama, Ibaraki 309-1793 Japan; 4 Department of Nephrology, Tsukuba Gakuen Hospital, Ibaraki, 2573-1 Kamiyokoba, Tsukuba, Ibaraki 305-0854 Japan; 5 Department of Nephrology, Ibaraki Clinical Education and Training Center, University of Tsukuba Hospital, 6528 Koibuchi, Kasama, Ibaraki 309-1793 Japan

**Keywords:** Autosomal dominant polycystic kidney disease (ADPKD), Systemic lupus erythematosus (SLE), Coronavirus disease 2019 (COVID-19), SARS-CoV-2 vaccination

## Abstract

Although SARS-CoV-2 vaccines, particularly mRNA-based formulations, have demonstrated high efficacy and safety, adverse events including autoimmune activity have been reported. We report a case of systemic lupus erythematosus (SLE) following SARS-CoV-2 vaccination in a 52-year-old Japanese female with autosomal dominant polycystic kidney disease (ADPKD). The patient presented with nephrotic syndrome and severe thrombocytopenia and fulfilled the following four criteria for SLE classification: positive antinuclear antibodies, positive anti-ds-DNA antibodies, renal involvement, and reductions in two blood cell lines (leukopenia and thrombocytopenia). The patient responded favorably to prednisolone therapy, although hydroxychloroquine was avoided because of a suspected allergic predisposition. This case underscores the potential for exogenous triggers such as vaccination to induce SLE, highlighting the need for vigilance in managing autoimmune responses, particularly in patients with chronic conditions such as ADPKD. Despite the patient’s history of ADPKD, no previous reports have linked this condition to post-vaccine SLE. Concurrent nephrotic syndrome in patients with ADPKD is rare, and the possibility of other treatable glomerular diseases should be considered when this is seen.

## Introduction

SARS-CoV-2 vaccines have demonstrated high efficacy in preventing Coronavirus Disease 2019 (COVID-19) and its deterioration. The mRNA vaccines developed by Pfizer/BioNTech (BNT162b2) and Moderna/NIH (mRNA-1273) show 95% and 94.1% efficacy, respectively, in preventing symptomatic COVID-19 after two doses [1–3]. These vaccines induce strong antibody responses and are effective in various age groups and populations. However, adverse effects have been reported, the most common of which are pain at the injection site (47%), fatigue (28.2%), and joint and muscle pain (23.1%) [4]; headache (15.7%) and fever (13.6%) are also frequently reported [5]. Serious but rare adverse events include cardiovascular complications such as thrombosis, stroke, and myocardial infarction [6]. Although uncommon, neurovascular complications have also been reported, including ischemic stroke, cerebral sinus venous thrombosis, and intracerebral hemorrhage [7]. Several cases of systemic lupus erythematosus (SLE) after SARS-CoV-2 vaccination have been reported; however, there are no reports of such cases with autosomal polycystic kidney disease (ADPKD) [8]. Here, we report a case of SLE after SARS-CoV-2 vaccination in a 52-year-old Japanese female with ADPKD.

## Case report

Twenty-two years before presentation, a 52-year-old Japanese female was diagnosed with ADPKD at another hospital based on her mother’s diagnosis; however, she did not attend follow-up visits or routine health checkups. Four years before presentation, she resumed nephrology consultations following the initiation of treatment for hypertension. At that time, her total kidney volume was measured at 1,200 mL. Three years previously, her annual kidney volume growth rate was 6.7%, and tolvaptan therapy was considered; however, this plan was postponed because of scheduling challenges during the COVID-19 pandemic. The patient was transferred to our hospital; at that time, she had blood urea nitrogen 15 mg/dL and serum creatinine (Cre) of 1.05 mg/dL without severe renal dysfunction, and urinary protein was mild at 0.15 g/gCre. She had been administered a second dose of the SARS-CoV-2 mRNA vaccine (COMIRNATY intramuscular injection, Pfizer–BioNTech) 1 month prior. After vaccination, she developed generalized urticaria and fever, which resolved within a few days. One month later, at a routine outpatient visit, her serum albumin was 2.7 g/dL and her urinary protein was 6.3 g/gCre, leading to nephrotic syndrome; her platelet count was 22,000 /µL, which was considered significantly decreased. The patient was admitted to the hospital the following day for further examination and treatment.

**Fig. 1:**
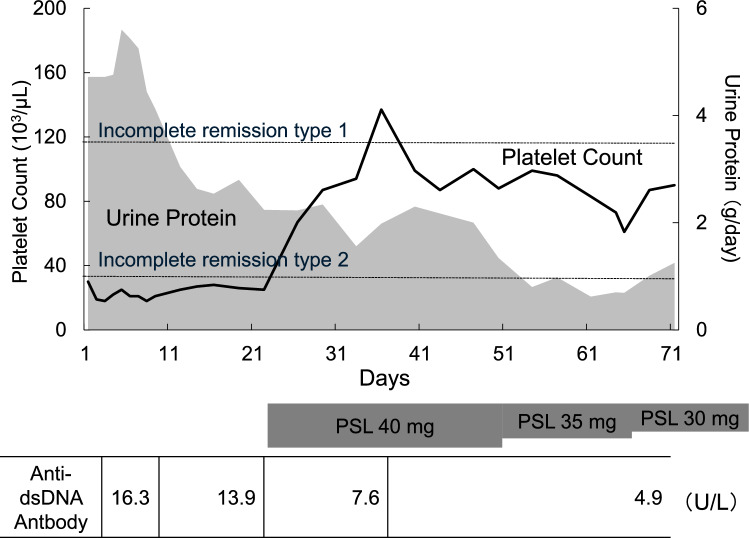
Clinical course of the patient. Urinary protein, platelet count and anti-dsDNA antibody titer after admission. The horizontal axis depicts the number of hospital days, left vertical axis represents the platelet count (bold line), and right vertical axis depicts urine protein (shaded). *PSL* Prednisolone

She had a history of hypertension, no history of smoking or alcohol consumption, and no specific allergies. She was taking an angiotensin receptor blocker (azilsartan) and a calcium channel blocker (nifedipine). Her mother had also been diagnosed with ADPKD, and her maternal aunt had undergone dialysis. She was obese (height: 153.2 cm, weight: 81.8 kg) and had a body mass index of 34.9. Her temperature was 37.1 °C, her blood pressure was 108/79mmHg, her heart rate was 93bpm, her SpO₂ was 98% (room air), and she had no vital signs of special note. Mild indurated edema was observed in both lower legs. Her palpebral conjunctivae were not pale, and there was no icterus of the bulbar conjunctivae. There was no cervical lymphadenopathy or tenderness. Thyroid enlargement was not observed. Breath sounds were clear and equal bilaterally. Heart sounds were regular, with no murmurs. The abdomen was flat and soft, with no tenderness. Laboratory results are presented in Table 1. Her hemoglobin was 8.4 g/dL, her platelet count was 30 000 /µL, and two types of blood cells (red and white) were decreased; serum albumin was decreased to 2.9 g/dL. Urine protein was 4 + using the dipstick method, with a high level of 7.64 g/gCre by quantitative analysis. Antinuclear antibodies were 1:160, and anti-DNA and anti-ds-DNA antibodies were positive; however, complement levels were not reduced. Figure 1 demonstrates multiple cysts in both kidneys on computed tomography (CT), and the total kidney volume was measured at 1633 mL.

**Table 1:** Laboratory data at hospitalization

Blood count		
White blood cell count	4900	/μL
Segmented Neutrophils	77.0	%
Band Neutrophils	0.0	%
Lymphocytes	11.0	%
Monocytes	7.0	%
Eosinophils	4.0	%
Basophils	0.0	%
Hemoglobin	8.4	g/dL
Hematocrit	25.6	%
Mean Corpuscular Volume	91.1	fL
Platelet Count	30,000	/μL
Segmented Neutrophils	77	%
**Biochemistry**		
Total Protein	6.8	g/dL
Albumin	2.9	g/dL
Aspartate Aminotransferase	36	U/L
Alanine Aminotransferase	22	U/L
Gamma-Glutamyl Transferase	60	U/L
Lactate Dehydrogenase	214	U/L
Total Bilirubin	0.9	mg/dL
Triglycerides	152	mg/dL
Low-Density Lipoprotein	128	mg/dL
C-Reactive Protein	0.9	mg/dL
Sodium	139	mEq/L
Chloride	105	mEq/L
Potassium	4.2	mEq/L
Calcium	7.8	mg/dL
Blood Urea Nitrogen	16.9	mg/dL
Creatinine	1.03	mg/dL
Uric Acid	7.5	mg/dL
Estimated Glomerular Filtration Rate	45.2	mL/min/1.73 m^2^
Ferritin	103.3	ng/mL
**Coagulation**		
Activated Partial Thromboplastin Time	58.8	sec
Prothrombin Time–INR	1.07	sec
D-dimer	2.7	μg/mL
**Urine Test**		
**Dipstick Urinalysis**		
pH	6.0	
Specific Gravity	1.009	
Glucose	–	
Ketones	–	
Blood	2 +	
Protein	4 +	
**Urine microscopy**		
Red Blood Cells	10–19	/HPF
White Blood Cells	5–9	/HPF
Hyaline Casts	0	/HPF
Granular Casts	0	/HPF
Red Blood Cell Casts	0	/HPF
White Blood Cell Casts	0	/HPF
**Urine Biochemistry**		
Protein	431	mg/dL
Creatinine	56.4	mg/dL
Protein to Creatinine Ratio	7.64	g/gCre
**Immunology**		
Immunoglobulin G	1950	mg/dL
Immunoglobulin A	244	mg/dL
Immunoglobulin M	277	mg/dL
Complement C3	90	mg/dL
Complement C4	20	mg/dL
50% Hemolytic Unit of Complement	51.1	U/mL
Antinuclear Antibody (HOMO)	160	fold
Anti-DNA Antibody	32	U/mL
Anti-dsDNA IgG Antibody	16.3	U/mL
Anti-Smith Antibody	< 1.0	U/mL
Lupus Anticoagulant	2.4	NR
Anti-Cardiolipin Beta2-Glycoprotein 1 Antibody	30.9	U/mL
Anti-Cardiolipin Antibody	83	U/mL
Platelet-Associated IgG Antibody	194	ng/107
ADAMTS13 Inhibitor	< 5.0	NR
Haptoglobin	178	mg/dL

*NR* normal range

Based on the 2019 European League Against Rheumatism/American College of Rheumatology criteria for SLE, the patient met the diagnostic threshold with a score of at least 27, exceeding the required minimum of 10 points. The entry criterion was fulfilled by a positive antinuclear antibodies titer (1:160). Her clinical features, including nephrotic-range proteinuria (6.3 g/gCre; 10 points), thrombocytopenia (platelet count 22,000 /µL; 4 points), and leukopenia (4 points), contributed significantly to diagnosis. Immunologically, her positivity for anti-dsDNA antibodies (6 points) further supported the diagnosis. She also fulfilled the Systemic Lupus International Collaborating Clinics criteria for SLE, meeting both clinical and immunological requirements. Although complement levels were normal, she met the minimum requirement of at least four criteria, including one clinical and one immunological criterion. A bone marrow examination was performed as a close examination for hemopenia; it showed only mild fibrosis, no obvious morphological or chromosomal abnormalities, and a mild increase in reactive juvenile blood cells and megakaryocytes, consistent with the findings associated with hemopenia due to SLE. Based on these findings, SLE was diagnosed and nephrotic syndrome was considered secondary to lupus nephritis.

**Fig. 2:**
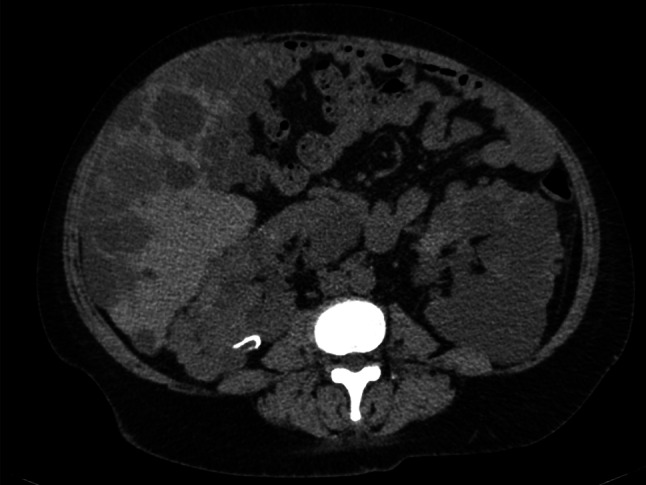
Computed Tomographic appearance of both kidneys. Non-contrast CT scan obtained at the time of admission reveals multiple cysts in both kidneys

After admission, she was placed on a low-salt diet and bed rest, and her urinary protein levels decreased to the equivalent of incomplete remission type II by the 12th day of hospitalization (Fig. 2). Treatment for SLE with oral prednisolone (40 mg/day) was initiated on day 23 with titrations as needed. Her anti-dsDNA antibody titer decreased over time, and her platelet count recovered quickly after the start of treatment, reaching 90,000/µL. The prednisolone dose was reduced to 30 mg/day on the 51st day; on day 54, urinary protein levels decreased to the equivalent of incomplete remission type I.

**Fig. 3:**
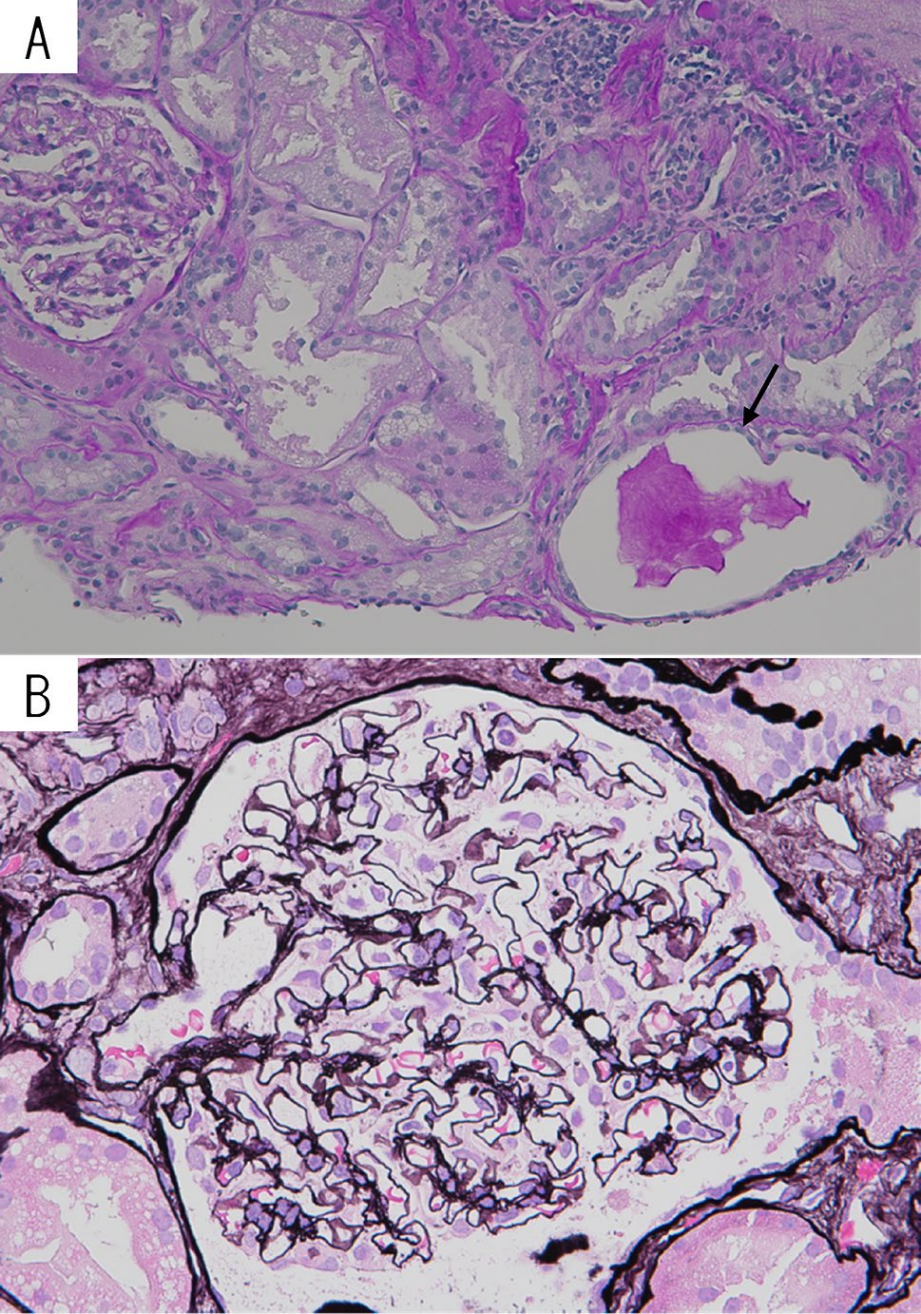
Light microscopic findings of renal biopsy. **A** Periodic Acid Schiff stain. The size of glomerulus was normal. A tubulus was dilated with hyaline cast in the lumen (arrow). **B** Periodic Acid-Methenamine silver stain

On the 64th day, retroperitoneoscopic renal biopsy was performed. Light microscopy revealed five glomeruli, of which two were globally sclerotic and two were collapsed. However, one glomerulus was preserved and available for evaluation (Fig. 3). This glomerulus showed no proliferative lesions in the mesangial area or extracapillary space, and there was no evidence of basement membrane duplication, spike formation, or a tram-track appearance. In the interstitial area, fibrosis and tubular atrophy were observed around the sclerotic glomeruli, accompanied by lymphocytic infiltration. Tubulitis or peritubular capillaritis was not identified. There was no fibrous intimal thickening of the renal vessels or arteriolar hyalinosis. In the specimens used for immunofluorescence and electron microscopy, glomeruli were not present. Immunofluorescence showed no significant interstitial immune deposits; electron microscopy revealed no tubulitis or peritubular capillaritis and no electron-dense deposits in the tubular basement membranes.

The prednisolone dose was reduced to 30 mg/day on the 65th day; her general condition was good and she was discharged home on the 71st day. After discharge, prednisolone was tapered off without recurrence of nephrotic syndrome, and the urinary protein level reached an equivalent level of complete remission on the 112th day.

## Discussion

To our knowledge, this is the first reported case of SLE occurring after SARS-CoV-2 vaccination in a patient with ADPKD. The patient was diagnosed with SLE after severe thrombocytopenia and nephrotic syndrome appeared one month after vaccination. Four of the SLE classification criteria were fulfilled, and the bone marrow findings were consistent with those associated with SLE. The patient responded well to the steroid treatment.

The age at which SLE most commonly develops in Japanese females is 20–39 years [9]; our patient was somewhat older (52 years), suggesting that SLE may have developed because of exogenous factors. Table 2 summarizes previous reports on SLE following SARS-CoV-2 vaccination. A case of a 60-year-old female who developed SLE after SARS-CoV-2 vaccination has been reported, suggesting that the SARS-CoV-2 vaccine may induce SLE exogenously regardless of age [10]. Cases of SLE after SARS-CoV-2 vaccination reported to date have developed SLE approximately 10–14 days after vaccination [8]. In our case, SLE was noted 1 month after vaccination, but the actual onset may have occurred earlier; therefore, we believe that there was no deviation from the course of previously reported cases.

**Table 2:** Summary of previous reports of systemic lupus erythematosus (SLE) following SARS-CoV-2 vaccination

Reference manufacturer	Age/sex	Vaccination timing	Treatment	Outcome
[10] N/A	60/F	Unknown	mPSL Pulse (15 mg/kg, 3 days), CPA IV 500 mg/biweekly, PSL 1 mg/kg, QD HCQ 100 mg BID	Decrease of urine protein
[18] Astra-Zeneca	23/F	First dose, 2 weeks before the onset	MMF, Glucocorticoids, HCQ (the exact dosages not specified)	Improvement of edema
[19] Moderna	27/F	Second dose, 2 weeks before the onset	HCQ 300 mg QD PSL 20 mg, MMF 2 g QD	Resistance to HCQ, decrease of urine protein after dose increase of PSL and MMF
[20] Pfizer	22/F	First dose, 1 week before the onset	mPSL Pulse, PSL 40 mg QD, HCQ 200 mg QD, AZA 50 mg	Decrease of urine protein
[21] Pfizer	24/M	Second dose, 2 weeks before the onset	PSL 1 mg/kg, MTX 15 mg/week	Improvement of symptoms (ex. Fever, arthralgia)
[22] Astra-Zeneca	22/F	First dose, 10 days before the onset	HCQ 400 mg QD PSL 50 mg, MMF 2 g BID	Improvement of symptoms (ex. pedal edema, petechiae, rash)
Our Case Pfizer	52/F	Second dose, 1 month before the onset	PSL 40 mg	Improvement of proteinuria and hemopenia

This table was partially modified from the table in [8] and created by including our case

*F* female, *M* male, *mPSL* Methylprednisolone, *CPA* Cyclophosphamide, *PSL* Prednisolone, *HCQ* Hydroxychloroquine, *MMF* Mycophenolate mofetil, *AZA* Azathioprine, *MTX* Methotrexate, *QD* Once daily (quaque die), *BID* Twice daily (bis in die)

The patient developed a skin rash and fever immediately after vaccination, suggesting an allergic predisposition. Hydroxychloroquine (HCQ), which has a relatively high frequency of drug-induced allergy, was discontinued, and the patient was treated with prednisolone alone. The patient responded well to steroid treatment, suggesting that allergic predisposition may be closely related to SLE pathogenesis. The patient relapsed after the reported course when HCQ was administered, causing urticaria and agranulocytosis refractory to antihistamines. HCQ is widely used as a first-line drug for SLE; however, in patients with SLE with a suspected allergic predisposition, as in our case, drug-induced allergy to HCQ should be carefully monitored.

Reports on the development of autoimmune diseases after vaccination against SARS-CoV-2 are scarce. The immunological background of patients with ADPKD differs from that of healthy controls, with increased inflammatory cytokines such as MCP-1 and TNF-α, macrophage infiltration, and up-regulation of immune checkpoint factors [11, 12]. Molecular mimicry of self-antigens by vaccines, activation of Toll-like receptors on antigen-presenting cells, and production of type I interferons have been proposed as mechanisms for the pathogenesis of SLE after vaccination [8, 13–17].

To date, three cases of lupus nephritis occurring in patients with ADPKD as an underlying condition have been reported [23–25], with our case being the fourth. Two of the previously reported cases involved women in their 40s, both diagnosed with lupus nephritis (one was class IV and the other was class V) and presenting with nephrotic syndrome. In contrast, the third case involved a woman in her 20s with lupus nephritis class II who did not exhibit nephrotic syndrome. Although none of the previous reports discussed the potential immunological predisposition of ADPKD in relation to the pathogenesis of SLE in detail, their coexistence has been considered coincidental [24]. Based on these reports, it is likely that there is little clinical association between ADPKD and the development of lupus nephritis. Since patients with ADPKD typically present with absent or minimal proteinuria, the occurrence of nephrotic syndrome or other signs suggestive of glomerular disease warrants consideration of a coexisting glomerulopathy that may require immunosuppressive therapy. In all reported cases, including ours, treatment with prednisolone or immunosuppressive agents resulted in a reduction in proteinuria.

In the present case, there were no clinical symptoms such as arthralgia or skin rash prior to vaccination. Additionally, laboratory tests performed at least 38 days before vaccination showed no cytopenia or massive proteinuria. Therefore, the manifestation of renal involvement and cytopenia due to SLE was considered to have occurred acutely. The development of generalized rash and fever after vaccination suggests that rapid activation of the immune system may have occurred, as described above. However, as immunological tests, including antinuclear antibody testing, were not performed before onset, the possibility that the patient had a predisposition to SLE prior to vaccination cannot be ruled out.

In conclusion, we reported the case of a 52-year-old female with ADPKD who developed SLE after SARS-CoV-2 vaccination. It is difficult to conclude whether the coexistence of ADPKD is an immunological predisposition for the development of autoimmune diseases induced by vaccination. In cases of ADPKD presenting with nephrotic syndrome, it is necessary to consider the identification of other treatable glomerular diseases. 

## References

[1] Polack FP, Thomas SJ, Kitchin N, Absalon J, Gurtman A, Lockhart S, et al. Safety and efficacy of the BNT162b2 mRNA Covid-19 vaccine. N Engl J Med. 2020;383(27):2603–15. 10.1056/NEJMoa2034577. 33301246 10.1056/NEJMoa2034577 PMC7745181

[2] Jackson LA, Anderson EJ, Rouphael NG, Roberts PC, Makhene M, Coler RN, et al. An mRNA vaccine against SARS-CoV-2—preliminary report. N Engl J Med. 2020;383(20):1920–31. 10.1056/NEJMoa2022483. 32663912 10.1056/NEJMoa2022483 PMC7377258

[3] Golob JL, Lugogo N, Lauring AS, Lok AS. SARS-CoV-2 vaccines: a triumph of science and collaboration. JCI Insight. 2021;6:9. 10.1172/jci.insight.149187. 10.1172/jci.insight.149187 PMC8262277 33822773

[4] Ganesan S, Al Ketbi LMB, Al Kaabi N, Al Mansoori M, Al Maskari NN, Al Shamsi MS, et al. Vaccine side effects following COVID-19 vaccination among the residents of the UAE-an observational study. Front Public Health. 2022;10: 876336. 10.3389/fpubh.2022.876336. 35602146 10.3389/fpubh.2022.876336 PMC9120526

[5] Li Y, Li J, Dang Y, Chen Y, Tao C. Adverse events of COVID-19 vaccines in the United States: temporal and spatial analysis. JMIR Public Health Surveill. 2024;10: e51007. 10.2196/51007. 39008362 10.2196/51007 PMC11287098

[6] Yasmin F, Najeeb H, Naeem U, Moeed A, Atif AR, Asghar MS, et al. Adverse events following COVID-19 mRNA vaccines: a systematic review of cardiovascular complication, thrombosis, and thrombocytopenia. Immun Inflamm Dis. 2023;11(3): e807. 10.1002/iid3.807. 36988252 10.1002/iid3.807 PMC10022421

[7] Panos LD, Bargiotas P, Hadjigeorgiou G, Panos GD. Neurovascular adverse effects of Sars-Cov-2 vaccination. Drug Des Devel Ther. 2024;18:1891–905. 10.2147/dddt.S464394. 38836116 10.2147/DDDT.S464394 PMC11147783

[8] Sagy I, Zeller L, Raviv Y, Porges T, Bieber A, Abu-Shakra M. New-onset systemic lupus erythematosus following BNT162b2 mRNA COVID-19 vaccine: a case series and literature review. Rheumatol Int. 2022;42(12):2261–6. 10.1007/s00296-022-05203-3. 36098769 10.1007/s00296-022-05203-3 PMC9468534

[9] Ohta A, Nagai M, Nishina M, Tomimitsu H, Kohsaka H. Age at onset and gender distribution of systemic lupus erythematosus, polymyositis/dermatomyositis, and systemic sclerosis in Japan. Mod Rheumatol. 2013;23(4):759–64. 10.1007/s10165-012-0733-7. 22903259 10.1007/s10165-012-0733-7

[10] Kim HJ, Jung M, Lim BJ, Han SH. New-onset class III lupus nephritis with multi-organ involvement after COVID-19 vaccination. Kidney Int. 2022;101(4):826–8. 10.1016/j.kint.2022.01.013. 35108572 10.1016/j.kint.2022.01.013 PMC8802143

[11] Kleczko EK, Marsh KH, Tyler LC, Furgeson SB, Bullock BL, Altmann CJ, et al. CD8(+) T cells modulate autosomal dominant polycystic kidney disease progression. Kidney Int. 2018;94(6):1127–40. 10.1016/j.kint.2018.06.025. 30249452 10.1016/j.kint.2018.06.025 PMC6319903

[12] Kleczko EK, Nguyen DT, Marsh KH, Bauer CD, Li AS, Monaghan MT, et al. Immune checkpoint activity regulates polycystic kidney disease progression. JCI Insight. 2023;8:12. 10.1172/jci.insight.161318. 10.1172/jci.insight.161318 PMC10371237 37345660

[13] Tudela P, Martí S, Bonal J. Systemic lupus erythematosus and vaccination against hepatitis B. Nephron. 1992;62(2):236. 10.1159/000187043. 1436323 10.1159/000187043

[14] Agmon-Levin N, Zafrir Y, Paz Z, Shilton T, Zandman-Goddard G, Shoenfeld Y. Ten cases of systemic lupus erythematosus related to hepatitis B vaccine. Lupus. 2009;18(13):1192–7. 10.1177/0961203309345732. 19880567 10.1177/0961203309345732

[15] Soldevilla HF, Briones SF, Navarra SV. Systemic lupus erythematosus following HPV immunization or infection? Lupus. 2012;21(2):158–61. 10.1177/0961203311429556. 22235047 10.1177/0961203311429556

[16] Ntouros PA, Vlachogiannis NI, Pappa M, Nezos A, Mavragani CP, Tektonidou MG, et al. Effective DNA damage response after acute but not chronic immune challenge: SARS-CoV-2 vaccine versus systemic lupus erythematosus. Clin Immunol. 2021;229: 108765. 10.1016/j.clim.2021.108765. 34089859 10.1016/j.clim.2021.108765 PMC8171000

[17] Sim TM, Ong SJ, Mak A, Tay SH. Type I interferons in systemic lupus erythematosus: a journey from bench to bedside. Int J Mol Sci. 2022;23:5. 10.3390/ijms23052505. 10.3390/ijms23052505 PMC8910773 35269647

[18] Zavala-Miranda MF, González-Ibarra SG, Pérez-Arias AA, Uribe-Uribe NO, Mejia-Vilet JM. New-onset systemic lupus erythematosus beginning as class V lupus nephritis after COVID-19 vaccination. Kidney Int. 2021;100(6):1340–1. 10.1016/j.kint.2021.09.009. 34560139 10.1016/j.kint.2021.09.009 PMC8455236

[19] Báez-Negrón L, Vilá LM. New-onset systemic lupus erythematosus after mRNA SARS-CoV-2 vaccination. Case Rep Rheumatol. 2022;2022:6436839. 10.1155/2022/6436839. 35186342 10.1155/2022/6436839 PMC8856802

[20] N AM, Saleh AM, Khalid A, Alshaya AK, Alanazi SMM. Systemic lupus erythematosus with acute pancreatitis and vasculitic rash following COVID-19 vaccine: a case report and literature review. Clin Rheumatol. 2022;41(5):1577–82. 10.1007/s10067-022-06097-z. 35175446 10.1007/s10067-022-06097-z PMC8852987

[21] Nune A, Iyengar KP, Ish P, Varupula B, Musat CA, Sapkota HR. The emergence of new-onset SLE following SARS-CoV-2 vaccination. QJM. 2021;114(10):739–40. 10.1093/qjmed/hcab229. 34450645 10.1093/qjmed/hcab229

[22] Patil S, Patil A. Systemic lupus erythematosus after COVID-19 vaccination: a case report. J Cosmet Dermatol. 2021;20(10):3103–4. 10.1111/jocd.14386. 34418261 10.1111/jocd.14386 PMC8661983

[23] Wan RK, Kipgen D, Morris S, Rodger RS. A rare cause of nephrotic syndrome in autosomal-dominant polycystic kidney disease. NDT Plus. 2009;2(2):136–8. 10.1093/ndtplus/sfn197. 25949310 10.1093/ndtplus/sfn197 PMC4421363

[24] Akinbodewa AA, Adejumo OA, Ogunsemoyin AO, Osasan SA, Adefolalu OA. Co-existing autosomal dominant polycystic kidney disease and nephrotic syndrome in a Nigerian patient with lupus nephritis. Ann Afr Med. 2016;15(2):83–6. 10.4103/1596-3519.179735. 27044732 10.4103/1596-3519.179735 PMC5402818

[25] Park JI, Lee H, An JN, Chin HJ, Kim S. Laparoscopic biopsy-proven lupus nephritis in autosomal dominant polycystic kidney disease. Kidney Res Clin Pract. 2012;31(3):192–5. 10.1016/j.krcp.2012.06.002. 26894026 10.1016/j.krcp.2012.06.002 PMC4716091

