# Correction: A case of autosomal dominant polycystic kidney disease with systemic lupus erythematosus developing after SARS‑CoV‑2 vaccination

**DOI:** 10.1007/s13730-025-01021-1

**Published:** 2025-07-15

**Authors:** Kensuke Miyauchi, Joichi Usui, Tatsuya Shimizu, Akihisa Hattori, Soichiro Nomura, Takanobu Higashi, Toshiaki Usui, Chie Saito, Hirayasu Kai, Kunihiro Yamagata

**Affiliations:** 1 Department of Nephrology, University of Tsukuba Hospital, 2-1-1 Amakubo, Tsukuba, Ibaraki 305-8576 Japan; 2 Department of Nephrology, Institute of Medicine, University of Tsukuba, 1-1-1 Tennodai, Tsukuba, Ibaraki 305-8575 Japan; 3 Department of Nephrology, Ibaraki Prefectural Central Hospital, Ibaraki, 6528 Koibuchi, Kasama, Ibaraki 309-1793 Japan; 4 Department of Nephrology, Tsukuba Gakuen Hospital, Ibaraki, 2573-1 Kamiyokoba, Tsukuba, Ibaraki 305-0854 Japan; 5 Department of Nephrology, Ibaraki Clinical Education and Training Center, University of Tsukuba Hospital, 6528 Koibuchi, Kasama, Ibaraki 309-1793 Japan

**Correction: CEN Case Reports** 10.1007/s13730-025-00999-y

In this article, the captions to Fig. 1 and 2 were inadvertently swapped. The correct captions of Fig. 1 and 2 are given below.

**Fig. 1** Clinical course of the patient. Urinary protein, platelet count and anti-dsDNA antibody titer after admission. The horizontal axis depicts the number of hospital days, left vertical axis represents the platelet count (bold line), and right vertical axis depicts urine protein (shaded). *PSL* Prednisolone.

**Fig. 2** Computed Tomographic appearance of both kidneys. Non-contrast CT scan obtained at the time of admission reveals multiple cysts in both kidneys.

The original article has been corrected.

